# Does the public receive and adhere to boil water advisory recommendations? A cross-sectional study in Newfoundland and Labrador, Canada

**DOI:** 10.1186/s12889-015-2688-z

**Published:** 2016-01-05

**Authors:** Andria Jones-Bitton, Diana L. Gustafson, Kelly Butt, Shannon E. Majowicz

**Affiliations:** 1 Department of Population Medicine, University of Guelph, Guelph, ON N1G 2W1 Canada; 2 Division of Community Health and Humanities, HSC 2834 Faculty of Medicine, Memorial University, St. John’s, NL A1B 3V6 Canada; 3 School of Public Health and Health Systems, University of Waterloo, BMH 2316, Waterloo, ON N2L 3G1 Canada

**Keywords:** Boil water advisory, Public health communication, Water contamination, Guideline adherence, Drinking water

## Abstract

**Background:**

Highly publicized water supply problems highlight the importance of safe drinking water to the public. Boil water advisories (BWAs) are an important precautionary measure meant to protect public health by ensuring drinking water safety. Newfoundland and Labrador, Canada is a prime location for exploring public notification practices and adherence to recommendations as there were a total of 215 BWAs, affecting 6 % of the provincial population, in 145 communities between April 2006 and March 2007 when data for the present study were collected.

**Methods:**

Residents who received household water from a public water supply were randomly selected for a telephone interview. Collected data included participants’ notification of boil water advisory, satisfaction with information provided, and their adherence to recommendations.

**Results:**

Most participants learned that a BWA had been issued or lifted in their community through radio, television, or word of mouth. BWAs were issued for a range of operational reasons. Almost all participants who had experienced a BWA reported wanting more information about the reasons a BWA had been issued. Low adherence to water use recommendations during a BWA was common.

**Conclusions:**

This study is first to report on public adherence to boil water advisory recommendations in Canada. The findings raise public health concerns, particularly given the high number of BWAs issued each year. Further studies in partnership with community stakeholders and government decision-makers responsible for overseeing public water systems are needed to assess the perceptions of BWAs, the reasons for non-adherence, and to identify information dissemination methods to increase information uptake and public adherence with acceptable uses of public drinking water during a BWA.

## Background

Highly publicized water supply problems highlight the importance of safe drinking water to the public. Canadian examples include the *Escherichia coli* (*E. coli*) outbreak in Walkerton, Ontario in May 2000, the *Cryptosporidium* outbreak in North Battleford, Saskatchewan in April 2001, and the evacuation of Kashechewan First Nation in Fall 2005, when *E. coli* was discovered in the drinking water [1–3]. Less often published in the national press are smaller scale water contamination events and boil water advisories (BWAs), such as the years-long BWAs in many Aboriginal communities of Canada [4]. BWAs are an important precautionary measure meant to protect the public health by ensuring drinking water safety; however, they can also increase consumer anxiety and alter perceptions about public drinking water [5, 6].

In Newfoundland and Labrador (NL), 42 % of the approximately 500,000 residents live in rural areas, compared to 20 % of Canada’s overall population [7]. This province’s relatively small population is spread over a disproportionately large land mass with approximately 535 public water supply systems being maintained by communities [8]. Each year, there are an average of 200 BWAs in effect in NL, and long-term BWAs (lasting five or more years) are common [9]. Little is known about how NL residents take up and use information about BWAs or the extent to which they adhere with BWA recommendations. This lack of knowledge is also an issue nation-wide, as there are no published studies on public adherence with BWA recommendations in Canada.

In 2007, a mixed methods project funded by the Newfoundland and Labrador Centre for Applied Health Research and the Public Health Agency of Canada was undertaken to investigate public perceptions of public drinking water supplies in NL and adherence to water use recommendations during BWAs. Given the paucity of information of public adherence with BWAs in Canada, we analysed data from this study, as an initial exploration of BWAs in the province, to investigate: (1) how people learned that a BWA had been issued, and lifted, in their communities, (2) satisfaction with the information provided about the BWA, and (3) public adherence to BWA recommendations.

## Methods

A cross-sectional study using computer-assisted telephone interviewing was designed and conducted in March and April 2007 with NL residents that received their household water from a public water supply. The Human Investigations Committee at Memorial University approved the study.

The NL Department of Environment and Conservation provided a database that categorized all communities in NL by the type of water source with which they were supplied (i.e., public or private water supply system). Community names were then cross-referenced with the NL residential community telephone exchanges (Bell Aliant Regional Communications, L.P., St. John’s NL), and a list of community names with corresponding telephone exchanges for residences with public water supplies was created.

Professionally trained interviewers administered the questionnaire to a target sample of 500 participants. Telephone numbers were randomly selected from the sampling frame using a commercial database that excluded unlisted and “do not call” phone numbers (ASDE Survey Sampler Inc., Gatineau, Quebec). Phone calls were made every day of the week, and at various times throughout the day and evening to maximize response. The interview was conducted with the person who was identified as being most responsible for drinking water decisions in the household. Other inclusion criteria were: having a valid phone service at a residential household supplied by a public water source, being 18 years of age or older, and able to communicate in English. Each interview took an average of 20 min to complete. All participants were entered into a draw for a chance to win one of three $250 cash prizes.

The questionnaire was based on one used in a similar study [5], with modifications generated from focus groups on drinking water conducted with NL residents. Specifically, questions pertaining to BWAs were added, and the phrasing of some questions was modified to incorporate NL-specific vocabulary. The sections of the questionnaire relevant to the current paper included open- and closed-ended questions (e.g., yes/no, check all that apply, and a 5-point Likert scale question, where 1 = very unimportant to 5 = very important); some closed-ended questions also included open-ended response options to capture additional detail. Data were collected on whether participants had experienced a BWA, and where so, their experiences and behaviours during the BWA, as well as general demographic characteristics. Descriptive statistics were used to summarize the data, and Chi-square tests used to compare the demographics of the study population with the demographics of the general population of NL, with significance set at α < 0.05. All statistical analyses were conducted in StataMP, version 11.0.

## Results

### Response rate and study population

Of the 3424 telephone calls made, 2172 phone numbers were eligible (i.e., were valid numbers and not business or fax lines). Phone numbers were also excluded from the response rate calculation if they were unreachable after four or more call-back attempts. A total of 563 surveys were completed, resulting in an overall response rate of 25.9 % (563/2172). Of all participants who responded, 535 (95.0 %) answered the question pertaining to whether they had prior or current experience with a BWA. Approximately 63.7 % (341/535) reported having experienced a BWA in their community previously, and a further 2.8 % (15/535) said their community was currently under a BWA; hence, a total of 356 participants (66.5 % of total number of survey participants) had experienced or were currently experiencing a BWA at the time of the study, and thus make up the sample for the project described here. Not all questions were fully answered by all participants, so some analyses were conducted with smaller sample sizes, as noted.

Demographic characteristics of all participants, and of the subset of participants who reported previously or currently experiencing a BWA, were separately compared with the NL 2006 census population (Table 1). Compared to the census population, both groups of participants were more highly educated, had greater access to the Internet, and reported more individuals in the household. Women, individuals in the middle-income categories, and households with no children were over-represented; individuals in the 18–29 and 70 years and older age groups were under-represented.

**Table 1:** Demographic comparison of survey participants (March–April 2007) with the NL 2006 census population [21]

	Survey population^a^# (%)	BWA population^b^ # (%)	Census population # (%)
Gender			
Male	218 (38.7)	129 (36.2)	245,735 (48.6)
Female	345 (61.3)	227 (63.8)	259,735 (51.4)
Totals	563 (100.0)	356 (100.0)	505,470 (100.0)
Comparison with Census population	Χ^2^ = 22.04	Χ^2^ = 21.82	n/a
	*p* <0.0001	*p* < 0.0001	
Age Group (Years)			
18–29^c^	48 (8.6)	25 (7.1)	58,615 (14.9)
30–39	93 (16.7)	60 (16.9)	67,475 (17.2)
40–49	148 (26.5)	98 (27.7)	84,440 (21.5)
50–59	137 (24.5)	90 (25.4)	82,175 (20.9)
60–69	102 (18.3)	60 (16.9)	52,320 (13.3)
70+	30 (5.4)	21 (5.9)	48,110 (12.2)
Totals	558 (100.0)	354 (100.0)	393,135 (100.0)
Comparison with Census population	Χ^2^ = 56.90	Χ^2^ = 39.44	n/a
	*p* <0.0001	*p* < 0.0001	
Highest level of school completed			
Grade school	77 (14.3)	78 (22.0)	141,575 (34.4)
High school certificate or equivalent	137 (25.4)	84 (23.7)	93,330 (22.6)
College or technical school graduate	195 (36.2)	131 (37.0)	125,480 (30.5)
University graduate	92 (17.1)	41 (11.6)	47,690 (11.3)
Post-graduate degree	38 (7.0)	20 (5.7)	3615 (0.9)
Totals	539 (100.0)	354 (100.0)	411,690 (100.0)
Comparison with Census population	Χ^2^ = 316.20	Χ^2^ = 112.11	n/a
	*p* < 0.0001	*p* < 0.0001	
Household Income ($CAD)			
<10,000	14 (3.2)	6 (2.1)	9690 (4.9)
10,000 – 14,999	20 (4.5)	12 (4.3)	12,465 (6.3)
15,000 – 19,999	22 (5.0)	19 (6.7)	15,015 (7.6)
20,000 – 29,999	57 (12.9)	44 (15.6)	26,985 (13.7)
30,000 – 39,999	72 (16.3)	53 (18.8)	25,050 (12.7)
40,000 – 49,999	63 (14.3)	43 (15.3)	21,190 (10.8)
50,000 – 59,999	52 (11.8)	30 (10.6)	18,970 (9.6)
60,000 – 69,999	29 (6.6)	16 (5.7)	15,005 (7.6)
>70,000	112 (25.4)	59 (20.9)	52,810 (26.8)
Totals	441 (100.0)	282 (100.0)	197,180 (100.0)
Comparison with Census population	Χ^2^ = 21.86	Χ^2^ = 26.22	n/a
	*p* = 0.005	*p* = 0.001	
Number of people in household			
1	63 (11.3)	43 (12.1)	39,830 (20.2)
2	224 (40.1)	140 (39.6)	73,295 (37.2)
3	116 (20.8)	70 (19.8)	39,835 (20.2)
4	111 (19.9)	69 (19.5)	31,985 (16.2)
5	35 (6.3)	25 (7.1)	9370 (4.8)
6+	9 (1.6)	7 (1.9)	2875 (1.5)
Totals	558 (100.0)	354 (100.0)	197,190 (100.0)
Comparison with Census population	Χ^2^ = 30.73	Χ^2^ = 18.87	n/a
	*p* < 0.0001	*p* =0.002	
Mean number of people in household			
	2.75	2.75	2.5
Number of children in household			
0	378 (67.6)	235 (66.2)	61,955 (39.8)
1	85 (15.2)	58 (16.3)	48,820 (31.3)
2	72 (12.9)	44 (12.4)	35,105 (22.5)
3 or more	24 (4.3)	18 (5.1)	9865 (6.3)
Totals	559 (100.0)	355 (100.0)	155,745 (100.0)
Comparison with Census population	Χ^2^ = 181.4	Χ^2=^ 104.61	n/a
	*p* <0.0001	*p* < 0.0001	
Internet Access^d^			
Yes	428 (76.2)	249 (70.1)	308,842^e^ (61.1)
No	134 (23.8)	106 (29.9)	196,627 (38.9)
Totals	562 (100.0)	355 (100.0)	505,824 (100.0)
Comparison with Census population	Χ^2^ = 53.55	Χ^2^ = 12.20	n/a
	*p* <0.0001	*p* <0.0001	

^a^All survey participants

^b^Survey participants who had experienced or were currently experiencing a BWA

^c^Age range of comparison groups differ; sample: 18 – 29 years versus census: 20 – 29 years

^d^Comparison groups differ: sample conducted with individuals 18 years and older versus census data with individuals 16 years or older

^e^Count derived by taking reported proportion with internet access (61.1 %) and multiplying by total provincial population count (505,824)

### Boil water advisory notifications

Participants were asked to report how they learned that the BWA had been issued, and had been lifted, in their community from a list of different media for information dissemination (Table 2). The use of media varied; the highest proportions of participants reported radio, television and word-of-mouth.

**Table 2:** Media by which participants^a^ learned BWA had been issued and lifted (March – April 2007, NL)

Information media	BWA issued # (% of participants) *n* = 356	BWA lifted # (% of participants) *n* = 340
Radio	179 (50.3)	174 (51.1)
Television	99 (27.8)	88 (25.9)
Word of mouth	80 (22.5)	82 (24.1)
Poster at local business	52 (14.6)	41 (12.1)
Mail flyer delivered to home	48 (13.4)	32 (9.4)
Newspaper	27 (7.6)	29 (8.5)
Other	15 (4.2)^b^	14 (4.1)^c^
Don’t know	3 (0.8)	8 (2.4)
Totals^d^	503	468

^a^Who had experienced or were currently experiencing a boil water advisory (BWA) in their community

^b^“Other” responses included: phone call from town council or local government office (*n* = 9), from town council or local government office, but specific method described unclear (*n* = 3), sign posted on way into town (*n* = 1), letter in the mail (*n* = 1), “website” (*n* = 1)

^c^“Other” responses included: from town council or local government office, but specific method described unclear (*n* = 4), phone call from town council (*n* = 3), in-person from town council member (*n* = 1), participant phoned town council (*n* = 1), participant worked for town council (*n* = 1), sign posted on way into town (*n* = 1), hotline advisory (*n* = 1), unclear response (*n* = 2)

^d^Totals exceed the number of participants as multiple responses allowed

### Satisfaction with provision of information related to boil water advisories

Approximately 71 % (254/356) of participants reported being satisfied with the information provided to them concerning the BWA in their community. Approximately 25 % (89/356) and 4 % (13/356) reported being dissatisfied and not knowing whether they were satisfied with the information provided, respectively. These individuals were asked to provide reasons for their lack of satisfaction, and a total of 90 participants (88.0 %) did so in an open-ended response; of these, 10 participants provided more than one reason. The responses were coded, and included: no reason for the BWA was provided (46.7 %; 42/90); too little information surrounding the BWA provided (31.1 %; 28/90); information was not disseminated widely enough (24.4 %; 22/90); information was disseminated too slowly (10.0 %; 9/90); information was not explained well (5.6 %; 5/90); and information was not individualized/sent directly to them (2.2 %; 2/90). Further, one participant reported not trusting the purveyors of the message (1.1 %; 1/90); one felt conflicting information was provided (1.1 %; 1/90); and one heard via word-of-mouth but wanted the information from a more reliable source (1.1 %; 1/90). Overall, almost all participants (98.3 %; 350/356) who had experienced a BWA reported that it was important or very important to them that they receive more information on the reasons why BWAs are issued.

### Adherence to boil water advisory recommendations

Participants were asked to identify from a list, the household activities, if any, for which they boiled public tap water before use during a BWA. The proportion of participants that reported boiling tap water (and thus, adhering to BWA recommendations) varied by activity: drinking: 74.4 % (232/312); cooking: 74.3 % (249/335); brushing teeth: 56.3 % (191/339); making ice cubes: 56.5 % (170/301); mixing juice: 64.1 % (191/298); washing ready-to-eat fruit and vegetables: 61.5 % (203/330); and making baby formula: 47.0 % (94/200).

## Discussion

Between April 2006 and March 2007, the time period related to this study, there were 215 BWAs in NL, affecting 145 communities and over 31,000 people [8]. The BWAs were issued for the following reasons: residual chlorination problem (36.3 %), no disinfection system (25.6 %), broken system or no chlorine (10.7 %), operational problem in the distribution system (9.3 %), disinfection system that was turned off by the operator (8.8 %), and failed microbiological tests (8.8 %) [8]. The procedures for issuing a BWA in NL are proactive and conservative in terms of public health. A BWA is issued if there is any possibility of risk to the community; therefore, the number of BWAs may not indicate the actual water quality in any given community.

In NL, a BWA is defined as “long-term” if it has been in place for more than five years [10]. As of March 31, 2013, there were 140 long-term BWAs in effect in the province, many of which served as substitutes for adequate drinking water treatment [9]. Concerns with heavy reliance on BWAs have been raised and include message fatigue, creation of unnecessary public panic, loss of public confidence in the water system, and risk of the public not taking seriously future BWAs [6, 11]. Dawe outlines policy recommendations for an improved drinking water advisory framework to help increase drinking water safety and reduce the number of BWAs in NL [9].

Our participants reported receiving BWA information from multiple media, the most common of which were radio, television and word-of-mouth. Although a flyer delivered door-to-door was the medium most participants (89 %; 503/563) reported being most likely to use to access general information on drinking water (data not shown), only 12 % of our participants who had been issued a BWA reported receiving information on the advisory in this way. The World Health Organization underscores the importance of notifying individuals impacted by the BWA (including residents, workers and travellers) as soon as possible, and suggests a variety of routes, including: media releases via television, radio and newspapers; telephone, email and fax contact of facilities, community groups and local agencies; posting of notices in public locations; personal delivery; and mail delivery [12]. The media through which the majority of study participants first learned of BWAs in communities in the United Kingdom [13], United States [14], and Netherlands [15], were leaflets delivered to homes, television, word of mouth, and radio. Other studies highlight the importance of regular engagement with local media and community networks in order to effectively reach consumers from a variety of demographic backgrounds [16, 17]. Given the rapid growth of social media platforms, we hesitate to identify examples that will become less meaningful and relevant over time. Rather, we recommend consulting regularly with communities to ensure that the means of notification fit their needs and preferences.

Approximately 74 % of participants in this study reported being satisfied with the information provided to them during the BWA; however, nearly all participants reported that it was important to them that they receive more information on reasons for issuance of BWAs. A study in a community that had recently experienced a boil water event asked participants for advice about issuing future boil water notices [13]. Approximately 46 % wanted more information at the start of the event, including a description of the potential health effects, and 35 % wanted more information provided intermittently throughout the boil water notice. Further, recommendations were made to better accommodate the needs of the elderly, and persons with disabilities who may have more specific needs regarding or understanding a boil water notice. Finally, although a door-to-door flyer was positively received, participants in that study felt that a loud speaker could have more alerted residents in a timelier manner, and a billboard along a main street could provide updates of the BWA status [13]. Town hall meetings may also provide town officials with an opportunity to distribute information to a larger audience and answer individual questions, and could be used to elicit community-specific suggestions for future information transfer. In light of our findings, increased dissemination of the reasons for issuing BWAs and the potential health effects of non-compliance is recommended. This, in conjunction with the engagement of community members, particularly those having previously experienced a BWA, in the development of information dissemination protocols could help improve public knowledge of, and potentially adherence with, with future BWAs.

Potentially further complicating the issue of BWA clarity, provinces and territories in Canada govern their own drinking water regulations. As such, there is no national standard for the terminology or definition of a BWA. This may lead to confusion and misinterpretation of out-of-province media reports, which may report on issues or concerns that are not relevant to NL. Although not examined in this study, standardization of drinking water terminology and definitions may reduce confusion and increase comparability of provincial drinking water reports.

The majority of our survey participants had experienced a BWA in their community. During a BWA, NL provincial guidelines recommend boiling water for drinking, brushing teeth, cooking, washing fruits/vegetables, and making ice, coffee/tea, infant formula/cereal and juices [18]. The reported low adherence (47 to 74 % depending on activity) among our study participants is consistent with other literature [13, 15, 16, 19]. Reasons for non-adherence with boil water advice have been reported elsewhere to include “forgetting” a BWA was in effect, “not believing” the notification, misunderstandings of the advice provided, a perceived lack of personal threat of illness, and the inconvenience of adhering to the recommendations [13, 15]. Concerning is the potential for message fatigue; decreasing adherence with BWA recommendations has been associated with increasing frequency and duration of BWAs issuances [6, 11]. Adherence with BWAs was found not to be dependent on sex, age and presence of children in the household in a study in the Netherlands; however, participants were 138.6 times more likely to comply with the advice if someone else in the household was also complying [15]. Similarly, a study conducted in the United Kingdom also reported that adherence with BWA recommendations was independent of demographic factors [17].

Some authors report no association between adherence with the BWA recommendations and the type of media by which people learned of boil water notices [13, 15, 16], whilst another reported that participants who received BWA information from the radio were more likely to adhere to recommendations for brushing teeth and preparing food than participants who received the information from a leaflet from the water company [17]. BWA information sheets that included the rationale and boiling procedures were shown to increase adherence among residents of Missouri, USA [19]. Studies to assess the reasons for non-adherence with BWA recommendations in NL would be useful; the long-term nature of many BWAs in the province may be important in this regard. Also needed are a set of effective knowledge transfer mechanisms that will result in positive changes in public behaviour and the implementation of strategies to increase adherence with BWA recommendations.

### Limitations

The low survey response rate (25.9 %) may affect the generalizability of the results; this is common in most survey-based research. Johnson and Wislar argue that response rates per se may not be the best metric for evaluating the quality of results; instead, emphasis should be placed on consideration of nonresponse bias [20]. If participants differed significantly from non-participants with respect to information uptake and adherence with BWA recommendations, our results may be biased. While we were unable to assess nonresponder characteristics, there were some significant differences in participant demographic characteristics compared to the census population, as noted.

Questions pertaining to BWAs were asked of all survey participants who had ever experienced such an event and not just those who had recently experienced a BWA. There is therefore, the potential for recall bias, particularly where there were protracted times between data collection and the experience of a BWA. Unfortunately, it was not possible to link participants with the specific BWA they reported on given the nature of the data collection, so we are unable to comment on relationships between BWA adherence and duration of the advisory; this would be interesting to explore in future work. Further, participants may have overestimated adherence with BWA recommendations in order to provide socially desirable responses. Finally, the results of this study should be interpreted in light of fact that long-term BWAs are relatively common in NL.

## Conclusions

Almost all participants who had experienced a BWA reported wanting more information about why a BWA had been issued. Low adherence to BWAs was common and varied by water use activity. This may be particularly concerning given the high number of BWAs issued each year. The public health risk associated with non-adherence to BWA, especially in areas where these are more common, should be concerning to various levels of government who oversee and are responsible for the safety of public water systems. Further site-specific studies to assess the perceptions of BWAs and the reasons for non-adherence would be useful. Also recommended are partnerships with community members to identify information dissemination methods and other strategies to increase information uptake and public adherence with acceptable uses of public drinking water during a BWA.

### Availability of supporting data

Raw data supporting this study may be made available upon request to the corresponding author. As the original participant consent process did not explicate that raw data might be provided to others outside the original study team, access to raw data will only be considered after institutional ethics review by the requesting researcher/research team.

## Abbreviations

BWA: Boil water advisory
NL: Newfoundland and Labrador

## References

[1] Bruce Grey Owen Sound Health Unit (2000). Waterborne outbreak of gastroenteritis associated with a contaminated municipal water supply, Walkerton, Ontario, May-June 2000. Can Commun Dis Rep.

[2] Stirling R, Aramini J, Ellis A, Lim G, Meyers R, Fleury M (2001). Waterborne cryptosporidiosis outbreak, North Battleford, Saskatchewan, Spring 2001. Can Commun Dis Rep.

[3] Canadian Broadcast Corporation. Kashechewan: Water crisis in Northern Ontario, 2006. www.cbc.ca/news2/background/aboriginals/kashechewan.html Accessed 22 July 2015.

[4] Eggertson L (2008). Despite federal promises, First Nations’ water problems persist. CMAJ.

[5] Jones AQ, Dewey CE, Dore K, Majowicz SE, McEwen SA, Waltner-Toews D (2006). Drinking water consumption patterns of residents in a Canadian community. J Water Health.

[6] Grover R (2011). Boil, boil, toil and trouble: the trouble with boil water advisories in British Columbia [thesis].

[7] Statistics Canada. Rural Canada – Population, urban and rural, by province and territory (Canada), 2011. http://www.statcan.gc.ca/tables-tableaux/sum-som/l01/cst01/demo62a-eng.htm Accessed 22 July 2015.

[8] Government of Newfoundland and Labrador. Drinking water safety in Newfoundland and Labrador Annual Report, 2007. http://www.env.gov.nl.ca/env/waterres/reports/drinking_water/annual_report_2007.pdf Accessed 22 July 2015.

[9] Dawe PV (2013). Using quantitative microbial risk assessment to determine if health risk warrants boil water advisories in Newfoundland and Labrador: time for a new approach [thesis].

[10] Government of Newfoundland and Labrador. Drinking water safety in Newfoundland and Labrador Annual Report, 2012. http://www.env.gov.nl.ca/env/waterres/reports/drinking_water/annual_report_2012.pdf Accessed 22 July 2015.

[11] Hrudey SE, Hrudey EJ, Pollard SJT (2006). Risk management for assuring safe drinking water. Environ Int.

[12] World Health Organization. Guidelines for drinking-water quality, 3^rd^ ed. Volume 1 – Recommendations, 2008. http://www.who.int/water_sanitation_health/dwq/fulltext.pdf Accessed 22 July 2015.

[13] O’Donnell M, Platt C, Aston R (2000). Effect of a boil water notice on behaviour in the management of a water contamination incident. Commun Dis Public Health.

[14] Ram P, Blanton E, Klinghoffer D, Platek M, Piper J, Straif-Bourgeois S (2007). Household water disinfection in hurricane-affected communities of Louisiana: implications for disaster preparedness for the general public. Am J Public Health.

[15] Karagiannis I, Schimmer B, de Roda Husman AM (2009). Compliance with boil water advice following a water contamination incident in the Netherlands in 2007. Euro Surveill.

[16] Rundblad G, Knapton O, Hunter PR (2010). Communication, perception and behaviour during a natural disaster involving a ‘Do Not Drink’ and a subsequent ’Boil Water” notice: a postal questionnaire study. BMC Public Health.

[17] Rundblad G, Knapton O, Hunter PR (2014). The causes and circumstances of drinking water incidents impact consumer behaviour: comparison of a routine versus a natural disaster incident. Int J Environ Res Public Health.

[18] Government of Newfoundland and Labrador. Drinking water manual – bacteriological water quality, public and private water supplies, 2012. http://www.health.gov.nl.ca/health/publichealth/envhealth/drinking_water_manual_1-8.pdf Accessed 22 July 2015.

[19] Angulo FJ, Tippen S, Sharp DJ, Payne BJ, Collier C, Hill JE (1997). A community waterborne outbreak of salmonellosis and the effectiveness of a boil water order. Am J Public Health.

[20] Johnson TP, Wislar JS (2012). Response rates and nonresponse errors in surveys. JAMA.

[21] Statistics Canada. Newfoundland and Labrador (Code10) 2006 Community Profiles – 2006 Census (Statistics Canada Catalogue no. 92-591-XWE), 2007. http://www12.statcan.ca/census-recensement/2006/dp-pd/prof/92-591/details/Page.cfm?Lang=E&Geo1=PR&Code1=10&Geo2=PR&Code2=01&Data=Count&SearchText=Newfoundland%20and%20Labrador&SearchType=Begins&SearchPR=01&B1=All&GeoLevel=PR&GeoCode=10 Accessed 22 July 2015

